# Cage effects on synaptic plasticity and its modulation in a mouse model of fragile X syndrome

**DOI:** 10.1098/rstb.2023.0484

**Published:** 2024-07-29

**Authors:** Rasa Volianskis, Camilla J. Lundbye, Gillian N. Petroff, David. E. Jane, John Georgiou, Graham L. Collingridge

**Affiliations:** ^1^ Lunenfeld-Tanenbaum Research Institute, Mount Sinai Hospital, Sinai Health System, Toronto, Ontario M5G 1X5, Canada; ^2^ Department of Physiology, University of Toronto, Toronto, Ontario M5S 1A8, Canada; ^3^ Hello Bio Limited, Cabot Park, Avonmouth, Bristol BS11 0QL, UK; ^4^ TANZ Centre for Research in Neurodegenerative Diseases, University of Toronto, Toronto, Ontario M5S 1A8, Canada

**Keywords:** Fmr1, LTP, STP, CFC, NMDAR PAM, littermate syndrome

## Abstract

Fragile X syndrome (FXS) is characterized by impairments in executive function including different types of learning and memory. Long-term potentiation (LTP), thought to underlie the formation of memories, has been studied in the *Fmr1* mouse model of FXS. However, there have been many discrepancies in the literature with inconsistent use of littermate and non-littermate *Fmr1* knockout (KO) and wild-type (WT) control mice. Here, the influence of the breeding strategy (cage effect) on short-term potentiation (STP), LTP, contextual fear conditioning (CFC), expression of *N*-methyl-d-aspartate receptor (NMDAR) subunits and the modulation of NMDARs, were examined. The largest deficits in STP, LTP and CFC were found in KO mice compared with non-littermate WT. However, the expression of NMDAR subunits was unchanged in this comparison. Rather, NMDAR subunit (GluN1, 2A, 2B) expression was sensitive to the cage effect, with decreased expression in both WT and KO littermates compared with non-littermates. Interestingly, an NMDAR-positive allosteric modulator, UBP714, was only effective in potentiating the induction of LTP in non-littermate KO mice and not the littermate KO mice. These results suggest that commonly studied phenotypes in *Fmr1* KOs are sensitive to the cage effect and therefore the breeding strategy may contribute to discrepancies in the literature.

This article is part of a discussion meeting issue ‘Long-term potentiation: 50 years on’.

## 1. Introduction

Fragile X syndrome (FXS) is the most common monogenic cause of inherited intellectual disability. It is caused by mutations in the *fragile X messenger ribonucleoprotein 1* (*FMR1*) gene on the X chromosome, which lead to transcriptional silencing of the gene and no production of the fragile X messenger ribonucleoprotein (FMRP) [1–11]. FMRP has been found to be important in synaptic plasticity, including long-term potentiation (LTP), the strengthening of synaptic connections. A variety of changes in LTP have been observed in *Fmr1* knockout (KO) mice, ranging from inhibition [12–52] to no effect [12,13,15,29,32,33,36,38,40,42,49,53–59] or even an increase [49,51,55,58,60,61] (see table 1). There are many possible reasons for these inconsistencies, including the induction protocol used, the age and strain of the mice and a number of environmental factors. One such potential *environmental* difference is whether a study uses *littermate* or *non-littermate* mice. In some experiments, wild-type (WT) and *Fmr1* KO mice are born to the same parents (WT father and heterozygous (HET) mother) and these littermates are kept in the same home cage after weaning [62,63]. Alternatively, separate colonies of WTs and KOs are bred from WT–WT pairs and KO–KO pairs, respectively, and housed separately as non-littermates.

**Table 1.** Summary of studies examining LTP phenotypes in littermate and non-littermate *Fmr1* KO mice. (Studies examining LTP in *Fmr1* KO mice were subdivided into three groups: (i) littermate, (ii) non-littermate mice, and (iii) undefined (breeding scheme could not be determined). Only the *Fmr1* KO and *Fmr1* KO2 (missing *Fmr1* mRNA) mouse models of FXS are included. The LTP genotype and brain area studied are shown. Abbreviations: mPFC, medial pre-frontal cortex; LA, lateral amygdala; DG, dentate gyrus; ACC, anterior cingulate cortex; LPP, lateral perforant pathway; MPP, medial perforant pathway; TA, temporoammonic; aud cortex, auditory cortex; MF, mossy fibre; PP, pairing protocol; β-AR, β-adrenoceptors; HFS, high-frequency stimulation; TBS, theta-burst stimulation; mGluR, metabotropic glutamate receptor; STDP, spike timing-dependent plasticity; TBSP, TBS pairing protocol; NOR, novel object recognition; C/A, commissural/associational; LFS, low-frequency stimulation.)

	LTP phenotype	studies	ref.	brain area	induction protocol; notes
littermate	increase	Pilpel *et al*. 2009^a^^,b^	[55]	CA1	PP (age- and protocol- dependent effects); *Fmr1* KO2
		Connor *et al*. 2011^a^	[58]	CA1	β-AR-dependent heterosynaptic plasticity
		Martin *et al*. 2023	[51]	Cerebellum	100 stimuli, 10 Hz; presynaptic LTP mediated by β-AR (low [Ca])
	no change	Godfraind *et al*. 1996	[53]	CA1	HFS
		Paradee *et al*. 1999	[54]	CA1	TBS
		Li *et al*. 2002^c^	[12]	CA1	TBS (brain area-dependent effect)
		Pilpel *et al*. 2009	[55]	CA1	PP (age-dependent) / TBS (protocol-dependent); *Fmr1* KO2
		Zhang *et al*. 2009^a^^,^ ^b^	[56]	CA1	HFS / *Fmr1* KO; littermate: *Fmr1*^+/−^ mother and *Fxr2*^−/y^ father
		Auerbach & Bear 2010	[57]	CA1	HFS
		Connor et al. 2011^a^	[58]	CA1	HFS-induced synaptic tagging and capture
		Franklin *et al*. 2014a^c^	[29]	CA1	HFS (hippocampal region-dependent effect)
		Bostrom *et al*. 2015^c^	[32]	CA1	HFS (hippocampal region-dependent effect)
		Ghilan *et al*. 2015^c^	[33]	CA1	HFS (hippocampal region-dependent effect)
		Martin *et al*. 2016^c^	[36]	mPFC	TBS (area dependent); *Fmr1* KO2
	reduction	Li *et al*. 2002^c^	[12]	cortex	TBS (brain area-dependent effect)
		Suvrathan *et al*. 2010	[20]	LA	HFS; mGluR-dependent LTP
		Eadie *et al*. 2012	[23]	DG	HFS
		Padmashri *et al*. 2013	[26]	cortex	chemical LTP
		Boda *et al*. 2014	[27]	CA1	TBS
		Franklin *et al.* 2014a^c^	[29]	DG	HFS (hippocampal region-dependent effect)
		Franklin *et al*. 2014b	[30]	DG	HFS
		Bostrom *et al*. 2015^c^	[32]	DG	HFS (hippocampal region-dependent effect)
		Ghilan *et al*. 2015^c^	[33]	DG	HFS (hippocampal region-dependent effect)
		Neuhofer *et al*. 2015	[35]	accumbens	STDP
		Martin *et al*. 2016^b^	[36]	mPFC	TBS (age-dependent effect); *Fmr1* KO2
		Yau *et al*. 2016	[37]	DG	HFS; Female *Fmr1* HET mice
		Feuge *et al*.2019	[41]	CA1	chemical LTP; no increase in mEPSC amplitude
		Hwang *et al*. 2022	[46]	CA1	PP; interneurons
		Martin *et al*. 2023^a^	[51]	cerebellum	100 stimuli, 10 Hz; presynaptic LTP mediated by β-AR (high [Ca])
non-littermate	increase	Brager *et al*. 2012	[60]	CA1	TBSP
		Routh *et al*. 2013	[61]	CA1	TBSP
		Borreca *et al*. 2023^a^	[49]	DG	HFS; 24 hour post NOR / protocol-dependent
	no change	Lauterborn *et al*. 2007^a^	[15]	CA1	TBS (protocol-dependent effect)
		Seese et al. 2012	[59]	CA1	TBS; reported 5 min post-induction
		Lundbye *et al*. 2018^b^	[38]	CA1	TBS (protocol-dependent effect)
		Wang *et al*. 2018^a^	[40]	DG	Optogenetic induction protocol; C / A pathway
		Banke & Barria 2020^b^	[42]	CA1	LFS; 3 Hz, 2 min (age dependent)
		Borreca *et al*. 2023^a^	[49]	DG	HFS; No NOR (protocol-dependent effect)
	reduction	Lauterborn *et al*. 2007^a^	[16]	CA1	TBS (protocol-dependent effect)
		Meredith *et al*. 2007	[17]	PFC	STDP; increased threshold
		Wilson & Cox 2007	[18]	Neocortex	HFS
		Lee *et al*. 2011	[21]	CA1	TBS
		Xu *et al*. 2012a	[24]	ACC	PP
		Xu *et al*. 2012b	[25]	ACC	PP
		Lundbye *et al*. 2018^b^	[38]	CA1	TBS (protocol-dependent effect)
		Martinez & Tejada-Simon 2018	[39]	CA1	TBS
		Wang *et al*. 2018^a^	[40]	DG	HFS/ pairing protocol; both MPP and LPP
		Banke & Barria 2020^b^	[42]	CA1	LFS; 3 Hz, 2 min (age-dependent effect)
		Ostrovskaya *et al*. 2020	[52]	CA1	TBS; Lower probability of late-LTP in P21 KOs (studied < 2 to 5 week)
		Seese *et al*. 2020	[43]	CA1	TBS
		Zhan *et al*. 2020	[44]	Cerebellum	TBS
		Ordemann *et al*. 2021	[45]	CA1 (TA)	TBS
		Jeon *et al*. 2022	[47]	CA1	TBS
		Borreca *et al*. 2023^a^	[49]	DG	HFS; 1 hour post NOR (protocol-dependent effect)
		Li *et al*. 2023	[50]	mPFC	TBS
undefined	no change	Larson *et a*l. 2005^b^	[13]	APC / CA1	TBS (age-dependent lack of effect; APC)
	reduction	Larson *et al*. 2005^b^	[13]	APC	TBS (age-dependent effect)
		Zhao *et al*. 2005	[14]	ACC/LA	PP
		Hu *et al*. 2008	[18]	CA1	PP; acute and cultured slices
		Shang *et al*. 2009	[19]	CA1	Chemical LTP
		Yun & Trommer 2011	[22]	DG	TBS
		Chen *et al*. 2014	[28]	ACC	TBS
		Yang *et al.* 2014	[31]	Aud cortex	HFS; mGluR dependent LTP
		Koga *et al*. 2015	[34]	ACC	LFS; 2 Hz, 2 min; Presynaptic LTP
		Monday *et al*. 2022	[48]	DG	25 Hz, 3X + d-APV; conditional KO of FMRP in Granule Cells

^a^ Diverging results within paper due to induction protocol difference.

^b^ Diverging results within paper due to age differences.

^c^ Diverging results within paper due to brain area differences.

Mice may be influenced by interactions with their siblings, with their parents or a combination of the two, all of which can contribute to differences in their development. In this study, the term ‘cage effect’ encompasses any difference between the environments of non-littermate and littermate mice, including any potential gene-environment (G×E) interactions. As denoted in the format—subject genotype^(maternal genotype)^—non-littermate *Fmr1* WT^WT^ and KO^KO^, and littermate WT^HET^ and KO^HET^ mice, were used to examine the consequences of the cage effect on LTP, fear memory, expression of *N*-methyl-d-aspartate receptor (NMDAR) subunits, and the modulation of synaptic plasticity by an NMDAR-positive allosteric modulator (PAM). Previous studies have demonstrated that certain FXS phenotypes can be influenced by whether mice are bred in a littermate or non-littermate fashion. Specifically, littermate *Fmr1* WT^HET^ mice (i.e. born to *Fmr1* HET mothers) show hyperactivity and social avoidance profiles that are more similar to those found in littermate and non-littermate KOs (KO^HET^ and KO^KO^, respectively), than to non-littermate WT (WT^WT^) mice [64,65]. These results suggest that behavioural phenotypes may be influenced by the littermate environment.

Studies of LTP in *Fmr1* KOs have used a relatively even split of littermate and non-littermate mice (table 1; littermate = 21, non-littermate = 19, undetermined = 9). For example, in the CA1 region of the hippocampus, there was a LTP reduction in 3 out of 15 studies using littermate mice compared with 9 out of 14 using non-littermate mice (table 1). It is important to note that there are other differences between these studies, such as the age of the mice or the strength of the induction protocol [15,38].

Besides synaptic plasticity, other measures are affected in *Fmr1* KO mice in an inconsistent fashion, including the expression of NMDAR subunits. Alterations in the total expression of various NMDAR subunits have been reported to increase [38,66,67], decrease [32,50] or not change [32,68,69] in *Fmr1* KO mice (table 2). Behavioural studies in *Fmr1* KO mice examining long-term memory, such as through contextual fear conditioning (CFC), have also diverged, with some studies reporting impaired fear memory [39,54,70–72] and others finding intact fear memory [73–77] (table 3).

**Table 2.** Summary of studies examining NMDAR expression in littermate and non-littermate *Fmr1* KO mice. (Studies examining NMDAR expression in *Fmr1* KO mice were subdivided into two groups: (i) littermate and (ii) non-littermate mice.)

	NMDAR phenotype	studies	ref.
littermate	increase	—	—
	no change	Bostrom *et al*. 2015	[32]
		Chatterjee *et al*. 2018	[68]
		Yau *et al*. 2018	[69]
	decrease	Bostrom *et al*. 2015	[32]
non-littermate	increase	Schutt *et al*. 2009	[66]
		Toft *et al*. 2016	[67]
		Lundbye *et al*. 2018	[38]
	no change	—	—
	decrease	Li *et al*. 2023	[50]

**Table 3.** Summary of studies examining CFC phenotypes in littermate and non-littermate *Fmr1* KO mice. (Studies examining CFC (only context, no cued or trace conditioning) in *Fmr1* KO mice were subdivided into two groups: (i) littermate and (ii) non-littermate mice.)

	CFC phenotype	studies	ref.	mouse (background); notes
littermate	no change	Dobkin *et al*. 2000	[77]	*Fmr1* KO (FVB-129 and C57BL/6-129)
		van Dam *et al*. 2000	[73]	*Fmr1* KO (C57BL/6J)
		Spencer *et al*. 2006	[44]	*Fmr1* KO (C57BL/6J); littermate: *Fmr1*^+/−^ mother and *Fxr2*^−/y^ father
		Uutela *et al*. 2012	[75]	*Fmr1* KO (C57BL/6J); littermate: *Fmr1*^+/−^ mother and BDNF^+/−^ father
		Nolan *et a*l. 2022	[76]	*Fmr1* KO (FVB-129)
	decrease	Paradee *et al*. 1999	[54]	*Fmr1* KO (C57BL/6J)
		Baker *et al*. 2010	[70]	*Fmr1* KO (albino C57BL/6J)
		Reyes *et al*. 2021	[72]	*Fmr1* KO2 (C57BL/6J)
non-littermate	no change	—	—	—
	decrease	Ding *et al*. 2014	[71]	*Fmr1* KO (C57BL/6J); male and female
		Martinez & Tejada-Simon 2018	[39]	*Fmr*1 KO (FVB-129)

Accordingly, the current study aimed to systematically determine whether variations in breeding design contribute to the offspring developing differences in synaptic plasticity (short-term potentiation (STP) and LTP), NMDAR subunit expression and contextual fear memory. Cage effects were examined through comparisons of non-littermate *Fmr1* WT^WT^ and KO^KO^ and littermate WT^HET^ and KO^HET^ mice. In addition, the effectiveness of a low-efficacy PAM of NMDARs, UBP714, at rescuing synaptic plasticity deficits was compared in KO^KO^ and KO^HET^ mice.

## 2. Methods

### (a) Animals

**Figure 1.**
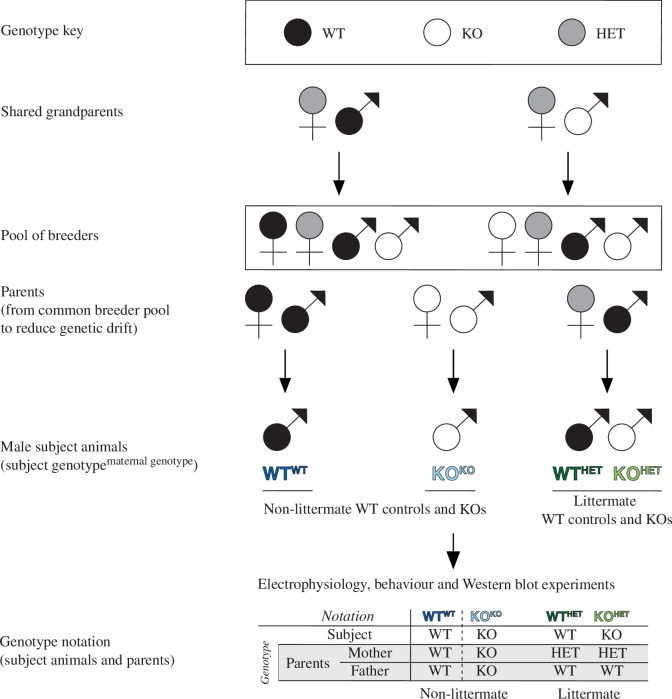
Schematic of the breeding paradigm used to generate mice of each experimental genotype^maternal genotype^ and cage background (littermate vs non-littermate). FXS is an X-linked disorder and, therefore, male mice only inherit their X chromosome from their mothers, whereas female mice inherit one X chromosome from each of the parents. A pool of breeders was created by crossing (i) a HET mother and WT father, and (ii) a HET mother with a KO father (shared grandparents). Using parents from the pool of breeders, male WT and hemizygous KO (males have one X chromosome) subject mice (experimental animals) were obtained; their genotype notation is shown together with their mother’s genotype in superscript (subject genotype^maternal genotype^). Non-littermate WT^WT^ mice were produced by crossing WT female and WT male mice, whereas for the non-littermate KO^KO^ mice, KO female mice were crossed with KO males. To obtain littermate WT and KOs, HET females were crossed with male WTs to produce WT^HET^ and KO^HET^ mice. These WT^WT^, KO^KO^, WT^HET^ and KO^HET^ mice were used for electrophysiological, behavioural and Western blotting experiments.

Experiments were performed at the Lunenfeld–Tanenbaum Research Institute (LTRI, Sinai Health System, Toronto, Ontario, Canada) and the Neurobehaviour Core at The Centre for Phenogenomics (TCP), according to an animal use protocol approved by the Animal Care Committee and conforming to the Canadian Council on Animal Care guidelines. Mice were group housed (maximum of five mice per cage) on 12 L : 12 D cycle (from 7.00), with food and water ad libitum. Female *Fmr1* KO mice [78] on a C57BL/6J genetic background (B6.129P2-Fmr1tm1Cgr; RRID:IMSR_JAX:003025) were further backcrossed with male C57BL/6J mice for three generations. To obtain male littermate WT and KO mice, female *Fmr1* HET mice were crossed with male *Fmr1* WT mice to produce WT^HET^ and KO^HET^ mice (subject genotype ^maternal genotype^). Male non-littermate WT^WT^ mice were produced by breeding female and male *Fmr1* WT mice while breeding female *Fmr1* KO with male hemizygous KO mice resulted in non-littermate KO^KO^ mice (breeding scheme and notation are summarized in figure 1). Note that breeders in both breeding strategies came from shared grandparents, thereby reducing genetic drift between the two groups. To study the cage effect (the differences, including GxE interactions, between littermate and non-littermate environments), male *Fmr1* mice were used and WT^WT^, KO^KO^, WT^HET^ and KO^HET^ mice were weaned at three weeks of age and housed with their littermates. All other aspects of the cages (dimensions, bedding and environmental enrichment) were identical between littermates and non-littermates. Mice aged between 8 and 14 weeks, with genotypes interleaved, were used for electrophysiology, CFC and biochemistry experiments. For each type of experiment, the same female researcher identified, handled and studied or collected each mouse (genotypes were not blinded). The number of animals is denoted as *N*, whereas the number of slices is shown as *n*.

### (b) Hippocampal slice preparation

Hippocampal slices were prepared as previously described [79]. Briefly, mice were decapitated under isoflurane anaesthesia and brains were removed and placed in cold (4°C) artificial cerebrospinal fluid (ACSF; all salts purchased from Wisent Inc., Canada) containing in mM: 130 NaCl, 10 d-glucose, 26 NaHCO_3_, 3.5 KCl, 1.2 NaH_2_PO_4_, 2 MgCl_2_ and 2 CaCl_2_ bubbled with carbogen (95% O_2_–5% CO_2_). Transverse dorsal hippocampal slices were prepared using a McIlwain tissue chopper and allowed to recover in room temperature ACSF for at least 2 h before electrophysiological recordings.

### (c) Electrophysiological recordings and data analysis

Hippocampal slices were transferred to a submerged chamber system (Scientifica, Uckfield, United Kingdom) and perfused at 2.5 ml min^-1^ with ACSF maintained at 30°C. Field excitatory postsynaptic potentials (fEPSPs) were evoked in the stratum radiatum of the CA1 area in response to bi-phasic stimulation (STG 4002; MCS, Multichannel systems, Germany) of Schaffer collateral/commissural (SCC) afferents with a platinum/iridium stimulation electrode (FHC, Bowdoin, ME, USA). Stimulation intensity was set to three times the threshold current required to evoke an fEPSP and baseline fEPSPs were recorded at 0.05 Hz. Recordings were amplified (Axopatch 1D, Molecular Devices, San Jose, CA, USA), digitized at 40 kHz (A/D) and recorded using WinLTP [80,81]. After 30 min of stable baseline, LTP was induced by theta-burst stimulation (TBS), consisting of four pulses at 100 Hz repeated five times at 5 Hz. In experiments with compounds, these were applied for 30 min prior to the induction of LTP.

Synaptic responses were analysed off-line to quantify changes in the initial fEPSP slope. To analyse STP and LTP, individual experiments were then normalized to their pre-induction baseline and fitted with a mono-exponential regression using Platin (a custom-built software package, Morten Skovgaard Jensen, Aarhus University, Denmark). Thus, the extent of LTP (%, the plateau) and amplitude of STP (%, maximum potentiation minus the plateau), and the decay time constant of STP (*τ* in min) were determined, as previously described [82,83]. It should be noted that STP comprises two mechanistically distinct components, STP1 and STP2, that have different *τ*-values [83]. However, with the current induction protocol, the level of STP2 is small, making it difficult to fit the datasets with double exponentials. Therefore, a single exponential fit was used as a weighted approximation. Furthermore, the assessment of STP ‘amplitude’ assumes that LTP has reached its plateau value at the time of the measurement. Since LTP may develop slowly [84], this value is likely to be an underestimate. Nevertheless, these measures provide a good approximation for the comparative assessment of synaptic plasticity changes between the groups of mice. Results are presented as averages of 2 min, normalized to baseline and plotted over time (mean values ± s.e.m.). The first minute after induction is excluded to reduce contamination of STP measurement by the short-lived, NMDAR-independent post-tetanic potentiation.

### (d) Pharmacology

UBP714 [85] was synthesized at the University of Bristol and was prepared in equimolar concentrations of NaOH to give a stock concentration of 100 mM. It was stored at −20°C and diluted in ACSF to the final concentration. The compound had no effect on baseline fEPSPs.

### (e) Contextual fear conditioning

CFC was performed using the Video Fear Conditioning System (Med Associates Inc., no. MED-VFC2-USB-M), and all experiments were performed at the same time of day (commencing at 13.00). Briefly, a mouse was placed in a CFC chamber (wiped with 70% isopropyl alcohol as an olfactory cue) and allowed to move freely for 3 min before receiving a 2 s, 0.75 mA, foot-shock. Each mouse was left in the chamber for a further 30 s and then removed into their home cage. At 24 h after conditioning, the mice were placed back into the CFC chamber with the same olfactory cue. No shocks were delivered and the amount of time the mice were immobile (% freezing) was measured over a 5 min period. The automated VideoFreeze™ Video Fear Conditioning Software (Med Associates Inc., Fairfax, VT) was used to analyse the behavioural data.

### (f) Western blotting

Hippocampi were extracted and kept in ice-cold ACSF. Under visual guidance using a stereomicroscope, whole dorsal CA1 chunks were isolated from both hippocampi of each mouse, pooled and placed on dry ice. Samples were then stored at −80°C until further tissue processing. Tissue was homogenized with a hand-held homogenizer in ice-cold radioimmunoprecipitation assay (RIPA) buffer (nos. 20–188, Millipore,) with protease/phosphatase inhibitor cocktail (no. 5872, Cell Signaling Technology). Samples were centrifuged at 15 000 × r.p.m. for 20 min at 4°C and the supernatant was collected. Protein concentrations were determined through the Pierce™ BCA Protein Assay Kit (no. 23228, Thermo Fisher Scientific, Canada) according to the manufacturer’s recommended protocol. Samples were denatured with 4 × Laemmli sodium dodecyl-sulfate (SDS) sample buffer (no. 1610747, BioRad) containing 2.5% β-mercaptoethanol (no. M3148, Sigma).

Protein samples (15 μg) were loaded on 7.5% TGX stain-free polyacrylamide gels (no. 1610181, BioRad) submerged in 1 × tris-glycine (TG)-SDS running buffer (no. 811–570-FL, Wisent). The gels were electrophoresed for 40 min at 200 V, before being activated and imaged using a ChemiDoc MP Imaging System (Bio-Rad Laboratories, Canada). Proteins were transferred to a low fluorescence polyvinylidene fluoride (PVDF) membrane (no. 1620264, Bio-Rad) using a wet transfer protocol in 1 × TG transfer buffer (no. 811–560-FL, Wisent) for 2 h at 35 V. Following transfer, total protein was imaged on the ChemiDoc system. Membranes were blocked using EveryBlot Blocking Buffer (no. 12010020, Bio-Rad) for 30 min at room temperature and incubated overnight with primary antibodies (GluN1: 1 : 5000 no. G8913, MilliporeSigma Canada; GluN2A: 1 : 5000 no. A0924, AbClonal Technology, USA; GluN2B: 1 : 2000, no. 66565-I-IG, Proteintech Group, USA) diluted in EveryBlot. Membranes were washed with 1 × tris-buffered saline Tween- 20 (TBST) and incubated for 1 h at room temperature with secondary antibody (StarBright SB520 no. 12005869, Bio-Rad or StarBright SB700 no. 12004158, Bio-Rad) diluted in EveryBlot at 1 : 2500 and 1 : 1500 dilution depending on the experiment. For multiplexing GluN2A and GluN2B targets, secondary antibodies with fluorochromes of different emission wavelengths were combined. Two-channel multiplex fluorescence images were collected on the ChemiDoc system.

Protein expression analysis was conducted using the Image Lab Software (version 6.1, Bio-Rad). Target protein levels were normalized to total protein, defined as the total adjusted lane volume of proteins between 37 and 250 kDa. The protein levels were expressed as a percentage of the WT^WT^ signal. Whole blots and total protein staining of targeted protein (i.e. GluN1, GluN2A and GluN2B) appear in the electronic supplementary material, figure S1.

### (g) Statistics

**Figure 2.**
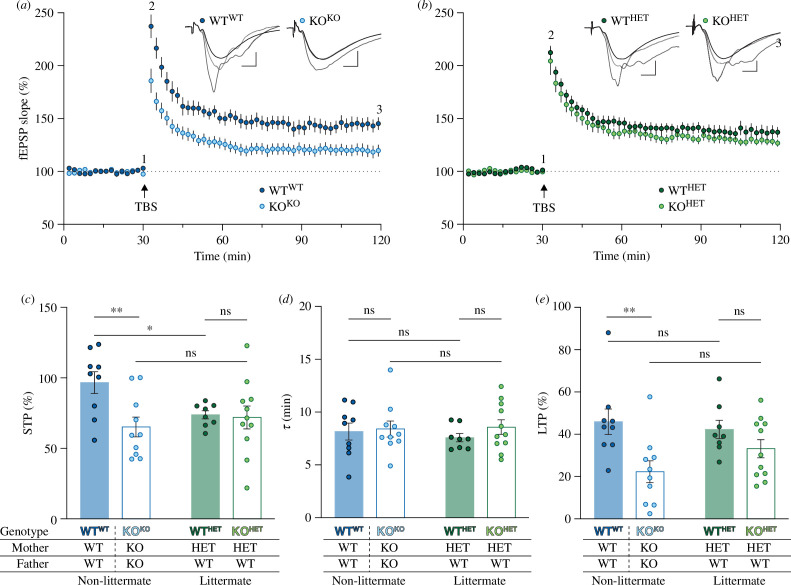
Breeding in a littermate or non-littermate fashion determines the synaptic plasticity deficit outcome in *Fmr1* KO mice. (*a*) Time course of synaptic responses (fEPSPs) in CA3-CA1 hippocampus synapses from non-littermate WT^WT^ (dark blue circles; *n* = 9) and KO^KO^ mice (light blue circles; *n* = 10). (*b*) Time course of fEPSPs in littermate WT^HET^ (dark green circles; *n* = 8) and KO^HET^ mice (light green circles; *n* = 11). Sample fEPSP traces (representative experiment) in the upper right sections of plots (*a*) and (*b*) are superimposed from (1) baseline, (2) STP and (3) LTP time points as indicated. Scale bar: 0.5 mV per 5 ms. (*c*) STP was affected by the genotype (*p* = 0.046), but not the cage effect (*p* = 0.269), with a significant interaction between the two variables (*p* = 0.026). Specifically, the extent of STP was significantly decreased in KO^KO^ and WT^HET^ mice compared with WT^WT^ mice (*p* = 0.004 and *p* = 0.039, respectively). There were no significant (ns) differences between slices from littermate KO^HET^ and WT^HET^ mice. *(d)* Neither genotype (*p* = 0.777) nor cage effect (*p* = 0.383; interaction, *p* = 0.596) influenced the decay time constant (* τ *) of STP. (*e*) The genotype (*p* = 0.003) but not the cage effect (*p* = 0.475; interaction, *p* = 0.158) had an effect on the level of LTP induced. Notably, there was only a significant deficit in non-littermate KO^KO^ mice compared with WT^WT^ mice (*p* = 0.002). Experiments were analysed with a two-way ANOVA with Fisher’s LSD post hoc test.

**Figure 3.**
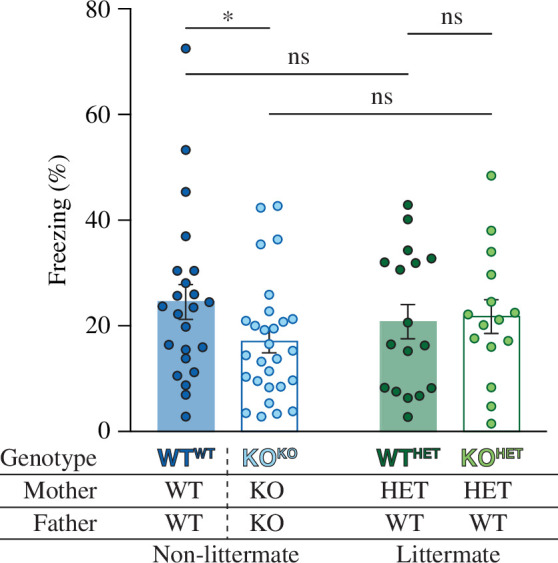
The cage effect determines fear memory deficit in *Fmr1* KO mice. At 24 h after contextual fear conditioning, mice were placed back into the conditioning chambers and their immobility (freezing) during the 5 min test was determined. The non-littermate KO^KO^ mice (open blue bar; *n* = 28) demonstrated deficits in freezing compared with WT^WT^ mice (filled blue bar; *n* = 23; *p* = 0.049). However, KO^HET^ mice (open green bar; *n* = 15) immobility was similar to their littermate WT^HET^ mice (filled green bar; *n* = 17; *p* = 0.840). Experiments were analysed with a two-way ANOVA with Fisher’s LSD post hoc test.

**Figure 4.**
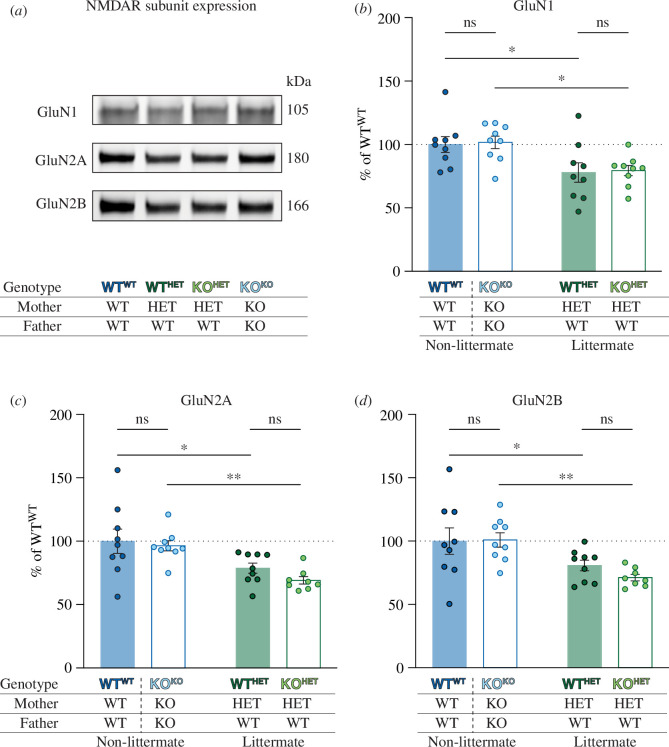
Differential expression of NMDAR subunits in the CA1 area of the hippocampus from littermate versus non-littermate *Fmr1* KO mice. (*a*) Representative blots of NMDAR GluN1, GluN2A and GluN2B subunits from hippocampus of WT^WT^, WT^HET^, KO^HET^ and KO^KO^ mice. Quantification of each targeted protein was normalized to total protein and expressed as a per cent of WT^WT^ (see the electronic supplementary material, figure S1 for full blots). (*b*) The cage effect (*p* = 0.001), but not genotype (*p* = 0.786; interaction, *p* = 0.983) influenced the expression of GluN1 subunits. WT^HET^ (filled green bar; *n* = 9) and KO^HET^ mice (open green bar; *n* = 9) had significantly decreased expression of GluN1 compared with WT^WT^ (filled blue bar; *n* = 9; *p* = 0.012) and KO^KO^ mice (open blue bar; *n* = 9; *p* = 0.012), respectively. (*c*) The decreased GluN2A expression in *Fmr1* KO mice was a result of the cage effect (*p* < 0.001) and not genotype (*p* = 0.269; interaction, *p* = 0.615). GluN2A expression in WT^HET^ mice (*n* = 9) was decreased compared with WT^WT^ mice (*n* = 9; *p* = 0.015) and expression in KO^HET^ mice (*n* = 8) was decreased compared with KO^KO^ mice (*n* = 9; *p* = 0.003). (*d*) GluN2B expression was not affected by the genotype (*p* = 0.515) but was affected by the cage effect (*p* = 0.001; interaction, *p* = 0.434). Specifically, expression of GluN2B was reduced in WT^HET^ mice (*n* = 9) compared with WT^WT^ mice (*n* = 9; *p* = 0.043) and in KO^HET^ mice (*n* = 8) compared with KO^KO^ mice (*n* = 9; *p* = 0.004). Experiments were analysed with a two-way ANOVA with Fisher’s LSD post hoc test.

**Figure 5.**
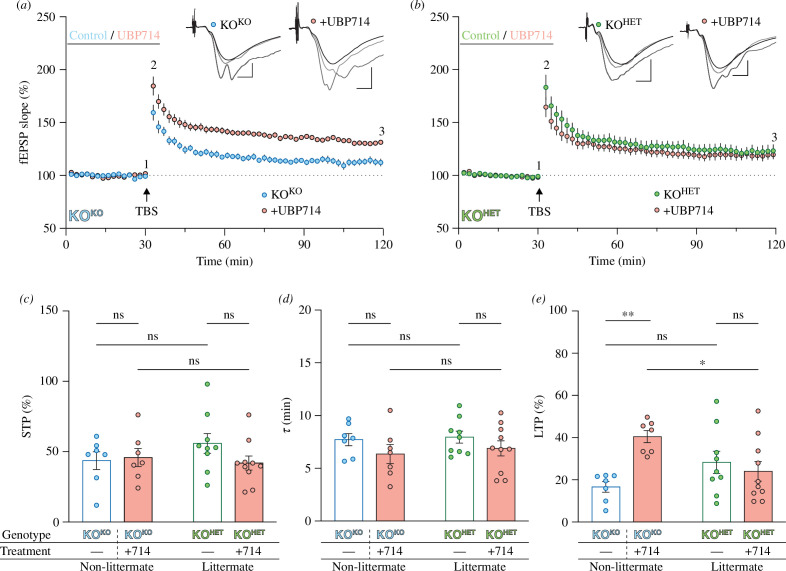
LTP in non-littermate KO^KO^ but not littermate KO^HET^, is sensitive to a NMDAR PAM. (*a*) Time course of fEPSPs in hippocampal slices from KO^KO^ mice, under vehicle (blue circles; *n* = 7) or UBP714 (100 μM; pink circles; *n* = 7) conditions. *(b)* Time course of fEPSPs in KO^HET^ slices, under vehicle (green circles; *n* = 9) or UBP714 (100 μM; pink circles; *n* = 10) conditions. Sample fEPSP traces (upper right section of plots in (*a*) and (*b*) are superimposed from (1) baseline, (2) STP and (3) LTP periods as shown. Scale bar: 0.5 mV per 5 ms. *(c)* Neither UBP714 application (*p* = 0.360), nor cage effect (*p* = 0.513) influenced STP (interaction, *p* = 0.211) in the two types of KO mice. *(d)* Similarly, the *τ* of STP was unaffected by the cage effect (*p* = 0.590) or UBP714 application (*p* = 0.097; interaction: *p* = 0.832). (*e*) Treatment with UBP714 had a significant effect on LTP (*p* = 0.035), with a significant interaction between UBP714 treatment and the cage effect (*p* = 0.004). The cage effect itself had no significant effect on LTP (*p* = 0.587). Specifically, the application of UBP714 to non-littermate KO^KO^ hippocampal slices significantly enhanced LTP (*p* = 0.001), while in the KO^HET^ slices UBP714 had no effect (*p* = 0.464). Notably, the level of LTP in KO^KO^ + UBP714 was significantly greater than in KO^HET^ + UBP714 slices (*p* = 0.012). Experiments were analysed with a two-way ANOVA with Fisher’s LSD post hoc test.

Statistical analysis was performed using GraphPad Prism. To analyse the influence of the parental genotypes and breeding schemes generating non-littermate and littermate mice, comparisons by two-way ANOVAs were applied to electrophysiology, CFC and Western blot data. A Fisher’s least significant difference (LSD) post hoc test was used for multiple comparisons to test the differences between: (i) WT^WT^ and KO^KO^, (ii) WT^HET^ and KO^HET^, (iii) WT^WT^ and WT^HET^, and (iv) KO^KO^ to KO^HET^ (figures 2–4). The cage effects and compound treatment on synaptic plasticity in the two KO groups were examined using a two-way ANOVA and differences between: (i) KO^KO^ and UBP714, (ii) KO^HET^ and UBP714, (iii) KO^KO^ and KO^HET^, and (iv) KO^KO^ + UBP714 and KO^HET^ + UBP714 were tested using Fisher’s LSD post hoc test (figure 5). The significance level was set to *p* < 0.05 and precise statistical values appear either within the text or figure captions, and post hoc results are denoted on the figures as *p < 0.05, **p < 0.01 and ***p < 0.001.

## 3. Results

To examine whether CA1 synaptic plasticity in male *Fmr1* KO mice is sensitive to the cage effect (see figure 1 for breeding scheme and nomenclature), TBS was delivered to the SCC to induce STP and LTP (figure 2; for all ANOVA statistics see the electronic supplementary material, table S1). When compared with WT^WT^ mice (96.7 ± 7.7%; *n* = 9), STP was significantly reduced in the KO^KO^ mice (65.2 ± 7.0%; *n* = 10; *p* = 0.004). Conversely, the level of STP in WT^HET^ mice (73.8 ± 2.9%; *n* = 8) and KO^HET^ mice (72.0 ± 8.1%; *n* = 11) was similar. The difference in the effects on STP can be attributed to a reduction in STP in the WT^HET^ compared with WT^WT^ mice (*p* = 0.039). In contrast to the cage effect on STP magnitude, the estimate of STP decay was similar between WT^WT^ (8.2 ± 0.8 min), KO^KO^ (8.4 ± 0.8 min), WT^HET^ (7.6 ± 0.4 min) and KO^HET^ (8.6 ± 0.7 min) mice. With respect to LTP, there was a breeding strategy-dependent effect. Thus, when compared with WT^WT^ mice (45.9 ± 6.0%), LTP was reduced in the KO^KO^ (22.3 ± 5.2%; *p* = 0.002). However, when compared with WT^HET^ mice (42.3 ± 4.3%), the level of LTP in the KO^HET^ mice (33.2 ± 4.3%) was not significantly different. In summary, with respect to both STP and LTP, there is a clear reduction in the KO^KO^ compared with the WT^WT^ groups but little or no difference between the between the WT^HET^ and KO^HET^ groups. Both the LTP and STP deficits show a cage effect and therefore are influenced by the breeding scheme.

CFC was carried out to test whether contextual fear memory is influenced by the cage effect (figure 3). Compared with WT^WT^ mice (24.6 ± 3.3%; *n* = 23), there was reduced freezing in the KO^KO^ mice (17.1 ± 2.1%; *n* = 28; *p* = 0.049). By contrast, the level of freezing in the WT^HET^ mice (20.8 ± 3.2%; *n* = 17) was similar to that in the KO^HET^ mice (21.7 ± 3.2%; *n* = 15). Neither the genotype nor the cage effect alone is sufficient to cause a deficit in contextual fear memory. Both the non-littermate environment and the lack of FMRP are necessary, leading to deficits in KO^KO^ mice compared with WT^WT^ mice.

To determine whether the expression of NMDARs relates to the differences in synaptic plasticity (figure 2) and fear memory results (figure 3), Western blots were carried out on GluN1, GluN2A and GluN2B subunits from CA1 hippocampus (figure 4). Irrespective of the breeding strategy, there were no significant differences between WT and KO mice in the expression of GluN1, GluN2A and GluN2B subunits. Interestingly, the expression of the three subunits was reduced in the littermate mice compared with the non-littermate mice. Thus, GluN1 expression, when compared with WT^WT^ mice (100.0 ± 6.2%; *n* = 9), was decreased in WT^HET^ mice (77.9 ± 7.7%; *n* = 9; *p* = 0.012); meanwhile, compared with KO^KO^ mice (101.7 ± 4.9%; *n* = 9), GluN1 was reduced in KO^HET^ mice (79.4 ±. 4.0%; *n* = 9; 0.012). Similarly, GluN2A expression was reduced in WT^HET^ mice (78.8 ± 4.0%; *n* = 9) compared with WT^WT^ mice (100.0 ± 9.6%; *n* = 9; *p* = 0.015) and in KO^HET^ mice (69.2 ± 2.9%; *n* = 8) compared with KO^KO^ mice (96.4 ± 4.0%; *n* = 9; *p* = 0.003). GluN2B subunit expression followed a similar pattern, with decreased expression in WT^HET^ mice (80.7 ± 4.2%; *n* = 9) compared with WT^WT^ mice (100.0 ± 10.4%; *n* = 9; *p* = 0.043), as well as in KO^HET^ mice (71.2 ± 2.6%; *n* = 8) mice when compared with KO^KO^ mice (100.9 ± 5.6%; *n* = 9; *p* = 0.004). As such, the cage effect appears to primarily be responsible for changes in the expression of NMDAR subunits.

Potentiation of NMDAR function using the PAM UBP714 enhances LTP in CA1 slices from WT rats and mice [79]. To examine whether this compound improves synaptic plasticity in the two types of *Fmr1* KOs, 100 µM UBP714 was applied for 30 min prior to TBS (figure 5). Treatment with UBP714 had no effect on baseline transmission or the magnitude of STP, with similar levels in KO^KO^ (43.7 ± 6.3%; *n* = 7), KO^KO^ + UBP714 (45.9 ± 6.4%; *n* = 7), KO^HET^ (55.9 ± 7.1%; *n* = 9) and KO^HET^ + UBP714 (42.0 ± 5.0%; *n* = 10) slices. Similarly, the decay time constant, *τ*, was similar in KO^KO^ (7.9 ± 0.5 min), KO^KO^ + UBP714 (6.4 ± 0.9 min), KO^HET^ (8.0 ± 0.6 min) and KO^HET^ + UBP714 (6.9 ± 0.7 min) slices. However, UBP714 enhanced LTP in the non-littermate KO^KO^ (from 16.7 ± 2.5% to 40.5 ± 2.9%; *p* = 0.001) but was ineffective in the littermate KO^HET^ (28.3 ± 5.3% and 24.0 ± 4.6%; *p* = 0.076). Thus, the effect of a NMDAR-PAM is also dependent on the cage effect.

## 4. Discussion

The current study investigated whether the cage effect (comprising differences between the environments or G×E interactions) of using male non-littermate, *Fmr1* WT^WT^ and KO^KO^, or littermate, *Fmr1* WT^HET^ and KO^HET^ mice (figure 1), influences synaptic plasticity, fear memory, protein expression and neuromodulation by a PAM, UBP714. It was found that all of these measures are sensitive to the cage effect.

### (a) Cage effects on long-term potentiation

The KO^KO^ mice had a substantially reduced LTP compared with WT^WT^ mice, while the KO^HET^ mice only showed a trend towards reduction of LTP compared with WT^HET^ mice. This effect was primarily caused by greater LTP in the KO^HET^ mice compared with the KO^KO^ mice. As such, the cage effect may ameliorate some of the LTP deficits observed in *Fmr1* KO mice in the CA1 area of the hippocampus. These results are consistent with the majority of studies carried out in the CA1, where studies using non-littermate *Fmr1* mice resulted in more frequently reported deficits in LTP than in littermate mice (table 1). It is known that environmental factors can influence LTP. For example, poor maternal care (low grooming by mothers) inhibits the induction of LTP in the offspring [86,87], and social defeat stress has been found to enhance LTP [88]. The majority of studies in other brain areas found a reduction of LTP regardless of whether littermate or non-littermate mice were examined (table 1), suggesting that LTP in area CA1 may be particularly sensitive to the cage effect.

### (b) Cage effects on short-term potentiation

Another form of synaptic plasticity, STP, was also examined in this study. STP, like LTP, is an increase in the strength of synaptic transmission following high-frequency stimulation, often delivered in bursts to mimic the endogenous theta rhythm. This increase in synaptic strength is transient and decays to either a stable LTP or back to baseline [89–91] in an activity-dependent manner [79,82]. While the role of LTP in long-term memory is commonly accepted, there is growing interest in STP as the potential mechanism for some forms of short-term memory [82,92–96]. STP has not been systematically examined in *Fmr1* KO mice. STP was substantially reduced in the KO^KO^ mice but was unaltered in the KO^HET^ mice when compared with WT^WT^ and WT^HET^ mice, respectively. The STP difference was principally owing to lower STP in WT^HET^ mice compared with WT^WT^ mice. Indeed, STP in WT^HET^ mice is similar to STP in both KOs. Interestingly, this STP pattern is similar to hyperactivity [64] and social avoidance phenotypes [65] in *Fmr1* mice, where the cage effect primarily influenced the WT^HET^ mice to be more like the KOs, rather than causing changes in the KO^HET^ mice. In summary, the breeding strategy influences the induction of STP.

### (c) Fear memory deficit is influenced by the cage effect

CFC is dependent on the hippocampus and, consistent with the LTP deficit, there was impaired CFC when KO^KO^ mice were compared with WT^WT^ mice but not when the two genotypes born to HET mothers were compared. As with LTP, environmental factors, such as poor maternal care [86] or exposure to social defeat [97], can influence memory in CFC. Studies examining CFC in *Fmr1* KO mice have yielded diverse results, with some studies reporting deficits in contextual fear memory [39,54,70–72], while other studies found normal levels of freezing [73–77]. The majority of studies have been carried out using littermate mice, with a relatively even split between those that found a deficit and those that did not. On the other hand, two non-littermate studies both showed impairments (see table 3). Collectively, these studies suggest that contextual fear memory in *Fmr1* KO mice is sensitive to the cage effect.

### (d) Expression of *N*-methyl-d-aspartate is sensitive to the cage effect

The cage effect also applied to the expression of NMDAR subunits, with reductions in GluN1, GluN2A and GluN2B subunits in both littermate WT^HET^ and KO^HET^ mice, compared with either WT^WT^ or KO^KO^ mice. KO^HET^ mice showed a trend towards a decrease in the expression of the GluN2 subunits compared with WT^HET^ mice, while WT^WT^ and KO^KO^ were similar in the expression of all three subunits. Previous studies have also reported variable effects on the expression of GluN subunits in FXS model mice (table 2). For example, a reduction in LTP has been associated with both an increase [ 37] and a decrease [31] in the expression of the GluN2A subunit. Studies in non-littermate mice showed either increased [38,66,67] or decreased [50] expression of NMDARs, whereas in littermate mice, the expression of NMDARs was either unaffected [32,68,69] or decreased [32] in the KO^HET^. As with LTP and CFC, environmental factors have been shown to affect the expression of NMDARs [87].

A surprising observation was that, compared with WT^WT^ mice, KO^KO^ mice had similar levels of expression of the NMDAR subunits even though STP, LTP and CFC were all reduced. Perhaps pertinent to this issue, the expression levels of the NMDAR subunits were measured in whole homogenate, which is a relevant parameter because FMRP is a translation regulator [98]. Total protein may not, however, reflect the synaptic density of these proteins, their functional state, receptor regulation or signalling.

### (e) *N*-methyl-d-aspartate modulation and cage effects

Ultimately, the goal of studying *Fmr1* KO mice is to understand the role of FMRP and to develop effective treatment strategies for FXS. Previous studies in *Fmr1* KO mice have found that modulation of specific NMDAR subunits could rescue deficits in LTP. Specifically, when increases in GluN2A subunit expression were found, an antagonist was able to reverse deficits in LTP [38], while when a reduction in GluN1 subunits was observed, d-serine and glycine could reverse deficits in LTP [32]. In the current study, the NMDAR was targeted using UBP714, a GluN2A/2B-preferring PAM. UBP714 was able to enhance LTP in the KO^KO^ but not the KO^HET^ mice; this may relate to the reduction in LTP in the KO^KO^ mice relative to the other three groups that showed more robust LTP. Consistent with this possibility, UBP714 was shown to potentiate LTP in WTs when a weak but not strong induction protocol is used [79]. Furthermore, UBP714 had no effect on STP in KO^KO^ and KO^HET^ mice, which is in line with previous results in WT mice [79]. In summary, the present findings demonstrate that environmental factors, such as the cage effect, may influence the effectiveness of neuromodulators in the *Fmr1* KO mouse model.

### (f) Do gene or environment interactions contribute to the cage effects?

FXS model phenotypes are influenced by the littermate environment. Specifically, littermate *Fmr1* WT^HET^ mice (i.e. born to *Fmr1* HET mothers) have been shown to be hyperactive and to display social avoidance profiles that are more similar to those found in littermate and non-littermate KOs (KO^HET^ and KO^KO^, respectively), than to non-littermate WT (WT^WT^) mice [64,65]. Indeed, the current study considered these cage effects can explain the range of inconsistent phenotypes that exist in the literature (tables 1–3).

It is now well-established that the sex of the experimenter can influence mouse stress, behaviours and responses [99–101], a confound that was minimized herein. To reduce other potential ‘human factors’, the fear memory freezing measures and the majority of the data analyses were automated. The current study was therefore designed to exhaust as many potential confounds as possible, and then to examine whether a range of parameters (synaptic plasticity, fear memory, NMDAR subunit expression and pharmacological rescue with a PAM) are influenced by the cage effect.

In comparisons between studies, the role of other factors, such as the mouse age or strain background, the induction protocol used, or the brain area examined, cannot be discounted. Furthermore, in our systematic study herein, the underlying factors leading to the cage effects on young adult mice are difficult to elucidate; the maternal or paternal care could vary between the breeding cages, there could be maternal programming effects, imprinting differences, the maternal microbiome could be influencing their offspring, or there could be other G×E interactions. Another possibility is the influence WT and KO littermate siblings have on each other. In terms of parental care, Zupan *et al*. [65] found no changes in grooming, arched back nursing in mothers, and time on nest in fathers between the groups [65]. Cross-fostering the WT^HET^ mice with WT mothers did not correct the social avoidance phenotype in the WT^HET^ mice. Furthermore, when they cross-fostered WT^WT^ mice with *Fmr1* HET mothers, they found that the WT^WT^ mice developed the social avoidance phenotype [65]. The authors suggest that both prenatal and postnatal exposure to the maternal HET environment is sufficient to induce changes in social behaviours [65]. As mentioned, another possibility is that the littermate siblings influence the phenotypes in each other. In the *Neuroligin3* KO mouse model of autism, the KO siblings influence the sociability phenotype of their WT littermates [102].

### (g) Concluding remarks

The current study has demonstrated that the cage effect of using littermate or non-littermate *Fmr1* KO mice differentially influences several phenotypes, including synaptic function, receptor expression, contextual fear memory and sensitivity to pharmacological rescue. The cage effect could explain some discrepancies in the literature on these and additional phenotypes. Overall, it is not known to what extent the littermate cage effect may be obscuring or enhancing phenotypes in mouse models of other disorders. Irrespective of the underlying causes, the present findings emphasize the need to carefully consider the type of breeding scheme used when studying FXS model mice and controls.

## Data Availability

Representative samples of Western blots and the raw electrophysiological field responses are included herein. Supplementary material is available online at [103].
